# Real-Time Processing Library for Open-Source Hardware Biomedical Sensors

**DOI:** 10.3390/s18041033

**Published:** 2018-03-29

**Authors:** Alberto J. Molina-Cantero, Juan A. Castro-García, Clara Lebrato-Vázquez, Isabel M. Gómez-González, Manuel Merino-Monge

**Affiliations:** Departamento de Tecnología Electrónica, ETS, Ingeniería Informática, Universidad de Sevilla, 41012 Sevilla, Spain; jcastro@dte.us.es (J.A.C.-G.); claralevaz@gmail.com (C.L.-V.); igomez@us.es (I.M.G.-G.); manmermon@dte.us.es (M.M.-M.)

**Keywords:** open-source hardware, digital signal processing, usability test, biomedical applications, Arduino

## Abstract

Applications involving data acquisition from sensors need samples at a preset frequency rate, the filtering out of noise and/or analysis of certain frequency components. We propose a novel software architecture based on open-software hardware platforms which allows programmers to create data streams from input channels and easily implement filters and frequency analysis objects. The performances of the different classes given in the size of memory allocated and execution time (number of clock cycles) were analyzed in the low-cost platform Arduino Genuino. In addition, 11 people took part in an experiment in which they had to implement several exercises and complete a usability test. Sampling rates under 250 Hz (typical for many biomedical applications) makes it feasible to implement filters, sliding windows and Fourier analysis, operating in real time. Participants rated software usability at 70.2 out of 100 and the ease of use when implementing several signal processing applications was rated at just over 4.4 out of 5. Participants showed their intention of using this software because it was percieved as useful and very easy to use. The performances of the library showed that it may be appropriate for implementing small biomedical real-time applications or for human movement monitoring, even in a simple open-source hardware device like Arduino Genuino. The general perception about this library is that it is easy to use and intuitive.

## 1. Introduction

In this work, we propose a software library, containing a set of classes, which allows the use of basic signal processing algorithms in low-cost open-source hardware (OSHW) platforms. The programmer will be able to set up simple biomedical applications very quickly and easily. Such applications include filtering out the power line interference in bio-electrical signals, smoothing, frequency analysis, etc. The software was written in C++ language and structured in several layers, which makes it flexible, helps separate different abstraction levels and allows good maintenance. Only one layer of the architecture is hardware dependent and contains the processes necessary to guarantee that data is sampled and delivered to other layers at a fixed rate. The programmer only needs to indicate the sampling frequency, number of analog channels and command this layer to start. The rest of the layers receive data from this one, like in a pipeline, where a task manager is in charge of executing the processes contained in them. A queue system interfaces with the layers, storing data temporally and giving more time for the different processes to be completed without losing data or interrupting the information flow.

We hypothesize that the code is reliable, easy to use and appropriate for most biomedical applications, even when using very cheap OSHW platforms. Therefore, our experiments were focused on showing the reliability and the accuracy of the results; its usability and the reasons that make this software easy to use and useful; and the software performances to demonstrate that it can be appropriate for the suggested applications.

Section 2 contains the state of art and describes several works in the area. In Section 3, the different parts of the proposed software architecture are explained in detail. Section 4 presents the experiments that were carried out and Section 5 outlines the results. Finally, Section 6 and Section 7 present a general discussion of the results, the conclusions and future work.

## 2. Background

Many devices in the environment around us, such as electrical appliances, automotive elements, etc., are controlled by small computers or micro-controllers which interface with sensors/actuators through digital or analog inputs/outputs. There is currently a growing interest in interconnecting these small computers to increase their interoperability and control through internal or external networks. This is called the Internet of Things (IoT) [1], which, in turn, is reinforcing the usage of these hardware platforms.

The specifications of these micro-computers (clock frequency, memory size, kind and number of peripheral devices such as timers, analog-to-digital converters (ADC), etc.) are set according to the number and type of input/output lines and the complexity of the algorithms running in them. In digital signal processing (DSP), most of the algorithms are based on the implementation of a finite number of sum-product operations that must be executed very quickly for real-time applications. The size of the data and its representation (floating or fixed) are also important for the accuracy of the results and must be taken into account in the selection of the hardware. For example, for audio processing, the data size should be equal to or greater than 16 bits with a sampling frequency of 44.1 kHz, (although some audio projects work at lower sampling frequencies, such as 32.25 kHz [2]), which gives a time interval of 22.7 μs to carry out all the processing in real time.

There are specific processors that speed up the execution of DSP algorithms by incorporating hardware such as 1-clock-cycle multipliers, barrel shifters, hardware loops, support for implementing circular buffering, multiple address and data buses to increase the bandwidth between the processor and the memory [3].

Several vendors offer digital signal processors and development kits for audio and video signal processing applications. It is even possible to find cheap and non-proprietary hardware platforms, aka Open-Source Hardware (OSHW) [4] or Libre Hardware that include these kinds of processors. In [5], for example, there are three boards (freeDSP, PiDSP and nanoDSP), costing around 60 €, based on the Analog Devices (AD) SimgaDSP processor familiy and containing up to 2-input/4-output analog channels. Users can easily implement audio processing algorithms through the SigmaStudio^™^ [6], a proprietary computer software which can be downloaded for free. Another OSHW platform can be found in [7], which is also based on the SigmaDSP processor’s family.

Libre Hardware is gaining significant traction in the scientific hardware community, where there is evidence that open development creates both technically superior and far less expensive scientific equipment than proprietary models, which have the option of manufacturing their own equipment [8]. Libre Hardware businesses already benefit from potentially lower costs, but there are several other advantages. By avoiding intellectual properties based licensing models (e.g., involving patents, lawyers, legal fees, lawsuits, etc.), Libre Hardware firms have substantially reduced legal fees compared to more conventional businesses. It is shocking that, today, many firms spend more on legal fees than engineering. For example, both Apple and Google spend more on legal fees than R&D [9].

**Figure 1:**
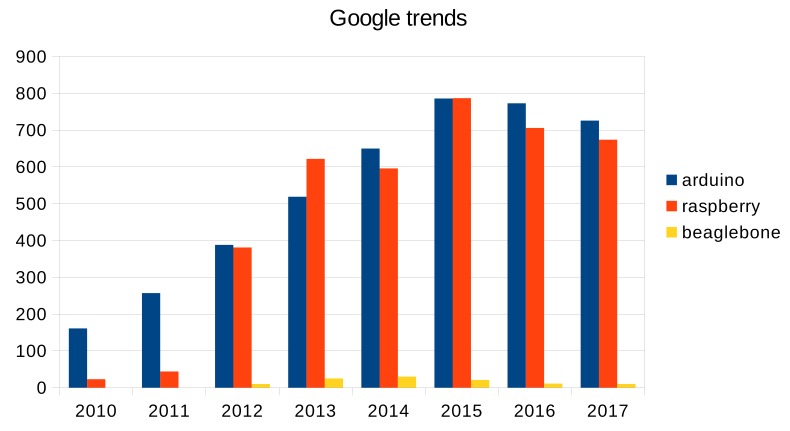
Google trends for Arduino, Raspberri Pi and Beaglebone boards in the last years.

Other popular OSHW platforms [10], but non specific for signal processing, include Arduino [11], RaspberryPi [12] and BeagleBoard [13]. RaspberryPi and BeagleBoard are based on ARM processors, while Arduino models are mainly based on Atmega processors. Some Arduino boards are also based on the ARM processor (e.g., Arduino Due). Arduino is better for beginners, for interfacing with external sensors or for battery powered applications [14]. In an embedded market study about integrating IoT and Advanced Technology Design [15], Arduino was the OSHW platform used most in current embedded designs (5.6%) or considered and being considered for use in the near future (17%), followed by Raspberry Pi (4.2% and 16%) and BeagleBone (3.4% and 10%). The interest in these boards is also supported by the searching trends in Google (Figure 1), where Arduino boards show slightly higher results than Raspberry Pi.

These platforms have been positively included in the electrical and electronic engineering studies [16] with a huge benefit to both curriculum and students. Students like the low cost of these devices and the ease of use that allows them to create significant projects, improve the system design and allow them to delve into real engineering systems motivated by their own creativity. Although there is no specific board for DSP in the Arduino family, one of the simplest ones (Arduino Uno), based on the 8-bit ATMega processor, has been used for teaching simple digital signal processing algorithms [17]. According to [18], Arduino platforms based on Atmega are not suitable for digital signal processing. However, they can work very well from an educational perspective because they allow more time to be spent on teaching fundamentals of DSP, and less on learning the integrated development environment, along with the support of a huge community [19]. Some studies benefit from easiness of the Arduino IDE and the supporting community to interface with Arduino with a shield containing DC/DC converters [20] or DSP processors for real-time digital signal processing [21,22,23,24].

Arduino boards have been used as single signal acquisition elements that deliver data to a computer where the digital signal algorithm is executed [25,26,27] or as processing units as well. Arduino boards containing 32-bit processors, such as Due or Uno32 have been used for different real-time applications: audio [28], finite or infinite impulsive impulse digital filters [29], image compressing [30] or for detecting R waves in electrocardiogram signals [31]. Nevertheless, boards based on 8-bit processors have been successfully used in biomedical applications such as cardio monitoring [32], online heart-rate detection [33], amputee rehabilitation [34], or for movement detection in people with disabilities based on the processing of signals delivered by accelerometers or flexometers [35]. As such applications do not need to process a large amount of data, the time constraints for real-time processing are more relaxed than in audio or video applications. Hence, this lets us consider using these single platforms for real-time DSP applications. Even for tiny audio applications, these single boards have been successfully used to make stereo audio output and a vocal effect device running at a rate of 44.1 kHz [36], some guitar effects [37] including echo, reverberation, etc. or tremolo [38] at a rate of 32.5 kHz. In [39], a project involving Arduino Uno to make a sound card can be found. The author develops a shield containing a digital-analog converter (DAC), a memory, which temporally stores incoming data, and amplifiers to adapt the voltage level of an input microphone and an output speaker. The project includes a set of routines that perform several sound effects like pitch up/down, delays, etc. at a rate of 44.6 kHz.

Implementing digital filters is an important issue in DSP. There are some available libraries including very simple filters. In [40], the filters are based on one- or two-pole analog designs for low/high pass filters. In [41], a low pass filter (1st and 2nd order, Chebychev and Bessel) and median filter are implemented. To change cutoff frequencies, the code needs to be reconfigured. In [29], the authors connect an Arduino Due to Matlab (Mathworks Inc., Natick, MA, USA) which sends up to 30 float-type coefficients for the implementation of the real-time filter. It has been shown that Arduino Due is suitable for audio application in real time although the developed program does not consider the samples to come at a fixed rate, which could affect the operation of the filter, especially when using infinite impulsive response (IIR) designs. In [39], the author implements a low pass finite impulse response (FIR) filter and a notch filter based on Arduino 8-bit processors. Although the author shows how to build a digital filter, its implementation cannot be considered as in real time and a fixed rate of sampling is not guaranteed. In [28,42], the flow of data is guaranteed at a fixed rate and both implement FIR filters for audio applications. The former implemented a 32-tap FIR filter with an execution time of 32 μs but based on an Arduino Uno32. The latter showed that the maximum length for a FIR filter in 8-bit-mono audio applications running at 31.25 kHz based on Arduino Uno was equal to 13.

Another important topic in DSP is frequency analysis, which is often done by applying the fast Fourier Transform (FFT). There is an Arduino library for computing the FFT algorithm [43] using blocks of data ranging between 16 and 256 words (16 b). The execution time of the FFT for the smallest block is 120 μs while for the largest one it is 7 ms. This means that, for an audio application running at a sample rate of 31.25 kHz (32 μs), the FFT algorithm could be executed in real time because it takes 256 × 32 μs ≈ 8.2 ms to fill a buffer with the incoming data and, then, compute the FFT. However, the memory requirements are very high because, according to the authors of the library, applying the FFT to a 256 word block of data requires 1 KB of memory, and to store the following block of data, while the FFT is computed, would need an additional 512 B of memory.For some simple Arduino models, like Arduino Uno, this means that 75% of the memory would be employed for the FFT computation for real-time processing in audio and there would be little memory left for the user application. Furthermore, while the FFT can be used for audio applications, this may be impractical due to the limitations of the block of data, which affects the frequency resolution $\Delta F=F_{s}/N$, where $F_{s}$ is the sampling rate, and N the block size. For example, with a sampling frequency of 32.25 kHz and a block size of 256 samples, the frequency resolution is equal to 122 Hz, which might be insufficient for certain types of applications.

Storing chunks of data before applying the DSP algorithm is a good practice and is necessary in some cases. On the one hand, it is mandatory for certain type of algorithms such as FFT, correlation, etc., and, on the other hand, it gives more time for the algorithm to be executed, relaxing the time constraints [28,42].

In this work, we propose a software architecture comprising a set of classes that allows programmers to easily and quickly make applications that include some DSP topics in 8-bit Arduino platforms. The main reason for starting with 8-bit platforms is the information we obtained from Google trends. The latest 2017 information shows that the interest in Arduino Uno accounted for 91% of searches in Google compared to 8% for Arduino Due and Uno32 together. The software architecture comprises several levels that allow it to adapt to other hardware platforms.

This software is an evolution of a previous work [35] where a five-layer software architecture for movement detection was proposed. That software contains low pass filters, guarantees sampling rate, includes a K-means algorithm and a finite state machine using software timers. Its main drawbacks are that the programmer needs to know the hardware, make use of low-level programming and that the software elements were not encapsulated, which, in turn, means that, for example, if two filters are needed, the code has to be repeated twice.

The DSP topics will include the capacity to process chunks of data or execute the algorithm sample by sample, guaranteeing a fixed sampling rate and offering FIR or IIR filters and frequency analysis based on FFT or Goertzel algorithm [44].

## 3. Software Architecture

One of the main goals of this work is to develop a software architecture wherein several processes, such as data acquisition, DSP algorithms and the user program can be independently executed as they receive data. To accomplish this, we propose structuring the software like a pipeline containing the processes in different layers. By segregating an application into layers, developers can modify or add a specific layer, creating flexible and reusable code, instead of having to rework the entire application. Several studies have included this multilayer architecture in their designs [35,45].

**Figure 2:**
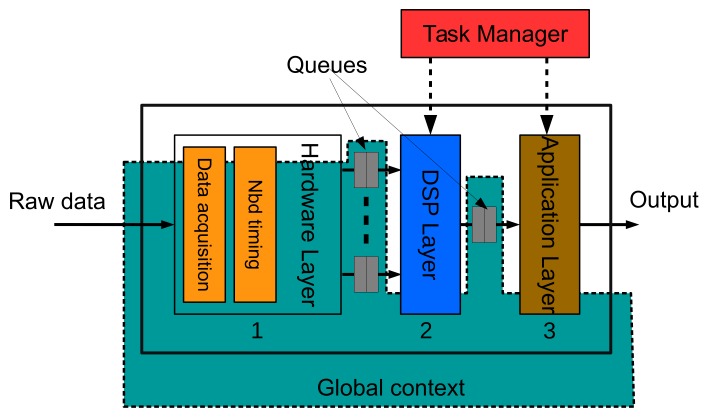
A processing pipeline containing three layers. The pipeline is managed by three main processes (orange and red in the figure). The data acquisition is a synchronous process that periodically samples data guaranteeing that they are delivered to higher layers in the pipeline at a fixed rate. The non-blocking timing is also an synchronous process that updates a list of timers. The third one, the Task Manager, is continuously executing the rest of the layer processes asynchronously. Finally, the global context contains the information that all processes share. The queues, timers and global variables are all defined in this area of the program.

To start with, Figure 2 shows a simple pipeline with three layers: hardware, DSP, and application. The pipeline shown is the example that we will follow in this paper to simplify explanations. There is a queue between two layers, storing data until the following data is able to read it. The hardware layer is the ‘heart’ of the pipeline. It contains a synchronous process that periodically samples the channels of the analog to digital converter (ADC) and dispatches the data to the following layer (DSP layer in our example) at a fixed rate through a set of queues. Each one of these queues, at this level, is associated with an ADC channel. The number of analog inputs or, in other words, the number of sensors that can be connected to the platform is limited by the hardware itself.

Another synchronous process in the hardware layer is in charge of updating the software timers that support the use of non-blocking delays as we will explain in detail later on in Section 3.1.2. The rest of the processes contained in DSP and application layers are sequentially executed by the Task Manager. The programmer decides the order of execution of each one. Processes in DSP and application layers are executed when the layer input queue contains new data or a timer object has expired. Following the guidelines of this software architecture, developers will be able to build more sophisticated pipelines including branches to parallel and longer lines.

The DSP layer contains a set of classes that allows programmers to implement typical digital signal processes like filters, basic frequency analysis and data segmentation (Section 3.2). The last layer, or the application layer, contains the end-user processes. Programmers should ensure they develop software that does not block the pipeline, assigns queue size appropriately to prevent data loss and allows the task manager to execute all the layers in less execution time than the time taken to process a block of samples. The usage of delay functions, long loops, etc. should be carefully considered in each layer. Finally, the architecture defines a global area containing the objects (queues, global variables, timers, etc.) that all processes share.

Algorithm 1 summarizes how to build the single three-layer pipeline. The hardware layer is not included because it is a periodic process performed by an interruption mechanism and it will be explained in the following section.

**Algorithm 1:** Pseudocode for implementing the proposed three-layer architecture in the example shown in Figure 2.
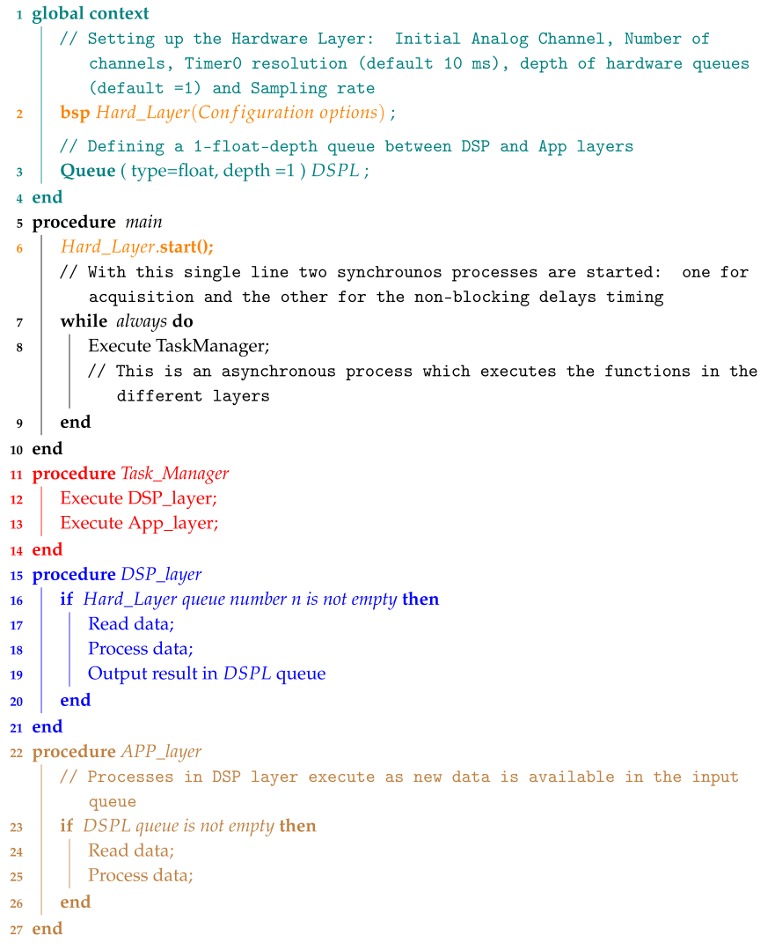


### 3.1. Hardware Layer

The hardware layer, HL, guarantees that samples are acquired at a fixed rate and supports the non-blocking delays (NBD). This layer acts as the ‘motor’ of the pipeline, periodically dispatching samples towards the following layer.

Two synchronous processes coexist in this layer: the data acquisition and the non-blocking timing processes.

#### 3.1.1. The Data Acquisition Process

**Figure 3:**
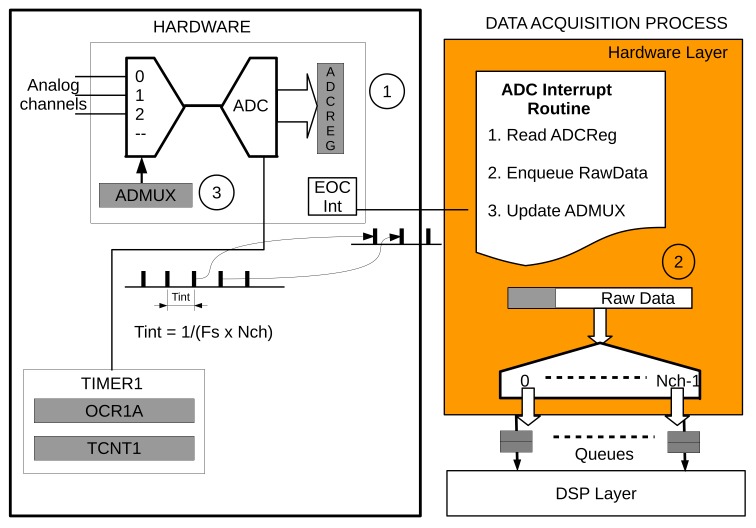
Timer1 is configured to trigger the Analog to Digital Converter, ADC, at a frequency rate of $F_{s}/N_{ch}$. The End-of-Conversion (EOC) interrupt (1) makes the ADC interrupt routine to read the data, sends it (2) to the Digital Signal Processing or DSP layer through the appropriate queue and (3) updates the ADC multiplexer, ADMUX, to select the next channel for conversion.

The processor’s Timer1 is configured to trigger the ADC at a rate equal to $T_{int}=1/\left(F_{s}\times N_{ch}\right)$, where $F_{s}$ is the sampling rate and $N_{ch}$ the number of channels. The End-of-Conversion (EOC) interrupt is executed when new data is available in the ADC. The interrupt subroutine reads the ADC, sends it to the following layer through the queue associated with the channel and selects the ADC channel for the following conversion. Figure 3 graphically depicts the processes involved in the data acquisition.

According to the manufacturer, the sampling rate at maximum resolution (10 b) is 15 kHz. Higher sampling rates are also possible, but with a reduced 8-bit resolution. In this work, only the maximum resolution scenario has been considered for the design of the library.

Another timer, Timer0, is configured to support the use of non-blocking delays, which, as happens with typical delay functions, wait for an established amount of time, but without blocking the execution of instructions or stopping the data flow in the pipeline. The timer class implements the non-blocking delays, and the timer_list class creates a list of pointers to timer objects. Timer0 makes interrupts to periodically access the list of timer pointers and decrease all the timer objects (see Section 3.3 for more details).

Additionally, this layer contains a set of routines to configure the micro-controller timers and the analog-to-digital converter. Programmers can configure the sampling rate and the number of channels used in this layer by changing certain variables in the code. Data is then automatically sent to the HL queues and are ready to be read by the next layer.

To create a Hardware Layer object, programmers only have to include two lines in their code: lines 2 and 6 shown in Algorithm 1.

#### 3.1.2. The NBD Timing Process

Delays are often used to make the processor wait for a timer to expire, as, for example, in the implementation of finite state machines. So as not to block the pipeline using traditional delays and prevent data from being lost, this software includes non-blocking delays. Two classes have been added to accomplish this goal.

The class timer includes two variables: *timeout*, which contains the actual timer value and *timeout initial*, which stores the initial value in case the timer object is restarted again. Several methods allow the timer to decrease, view its content, retrigger it or signal when the timeout has expired (*timeout* equal to zero). The timer class itself can be used as a counter/timer according to the rate of the process calling the decrease method. For example, if a timer interrupt periodically calls such a method, the timer class would be operating as a timer, otherwise it would work as a descent counter.

**Figure 4:**
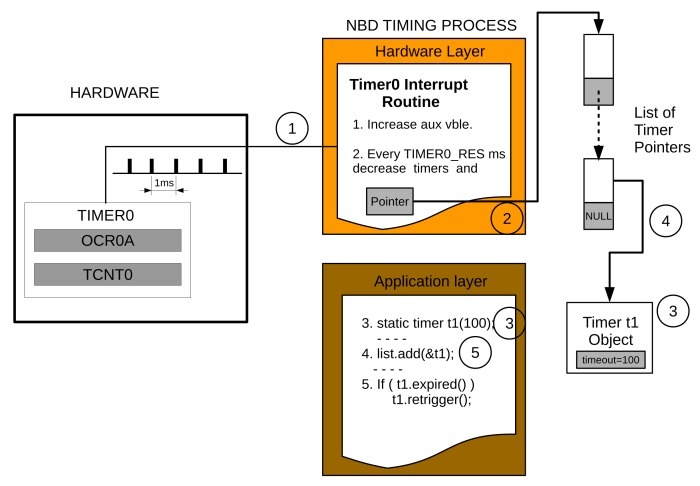
Support for non-blocking delays through Timer0 interrupts. Every 1 ms (1), an interrupt is generated, which makes an interrupt routine increase the **aux** variable. Every 10 ms (the default timer resolution), this routine searches for a list (4) of declared (3) and installed (5) timers in order to decrease their values. For example, Timer1 in the figure would expire after 1 s.

The Timer0 interrupt routine is called every 1 ms. Every TIMER0_RES seconds (the default Timer0 resolution is 10 ms) the routine searches for all installed timers in order to decrease them. Figure 4 depicts this procedure and Algorithm 2 contains a reduced code explaining how to use them.

**Algorithm 2:** Pseudocode explaining how to use non-blocking delays.
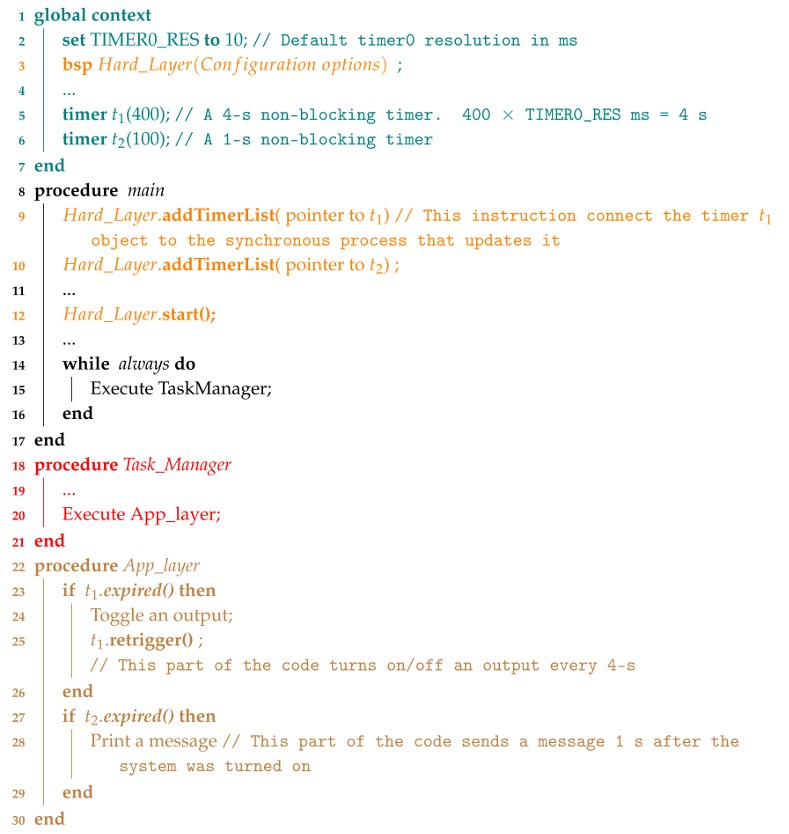


### 3.2. DSP Layer

If needed, this layer contains a set of basic digital signal processing applications for filtering and frequency analysis.

#### 3.2.1. Filter Class

This class allows programmers to implement digital filters [46] with p+1 and q+1 coefficients in the causal and anti-causal part of Equation (1), respectively:

(1) $$y\left[n\right]=\left(-\sum_{n=1}^{q}a\left[k\right]y\left[n-k\right]+\sum_{k=0}^{p}b\left[k\right]x\left[n-k\right]\right)/a\left[0\right].$$

The class has the following methods: *assign*, which allocates a buffer in memory with p+q+2 words to store the current and the q/p past inputs/outputs; the *reset* method empties the whole buffer and initializes the pointers; the *view* method provides access to the content of the buffer; and, finally, the *assess* method introduces the input data in the buffer using the modulo addressing technique [3] to reduce the execution time and evaluate the output of the filter.

The coefficients of the filter are given using the Q15 format [47], which ranges from roughly −1.0 up to 1.0. Q format numbers that are notionally fixed point numbers, that is, they are stored and operated upon as regular binary signed integers, thus allowing standard integer hardware/ALU to perform rational number calculations.

Computation of Equation (1) is performed using long words (32 b) to avoid overflows; then, the result is cast to an integer using the banker’s rounding that returns the even result if there are two nearest integers. This is good practice for IIR filters, which are very sensitive to rounding errors. Algoritm 3 shows an example using the filter class to implement a Butterworth low pass filter.

**Algorithm 3:** Simple example illustrating how to use the filter class.
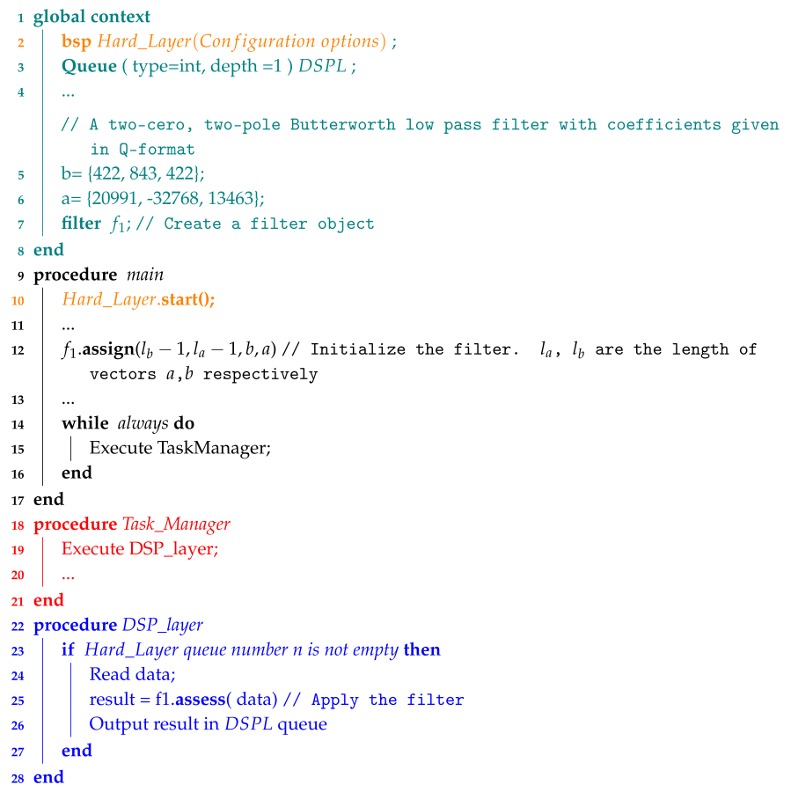


#### 3.2.2. Polyphasic Filter Class

This class implements a p-tap FIR polyphasic filter in decimation [48] that outputs results at a rate of $F_{s}/D$ (Equation (2). It is based on the filter class and its methods are fairly similar: *assign* and *assess*. The *assign* method allocates a buffer in memory with p+1 words and initializes the object and the *assess* receives data and outputs a result when available depending on the decimation factor:

(2) $$y\left[n\right]=\sum_{k=0}^{p}x\left[nD-k\right]h\left[k\right].$$

Algorithm 4 shows a segment of pseudocode illustrating how to include a polyphasic filter in a program.

**Algorithm 4:** Programming example illustrating the usage of pfilter class.
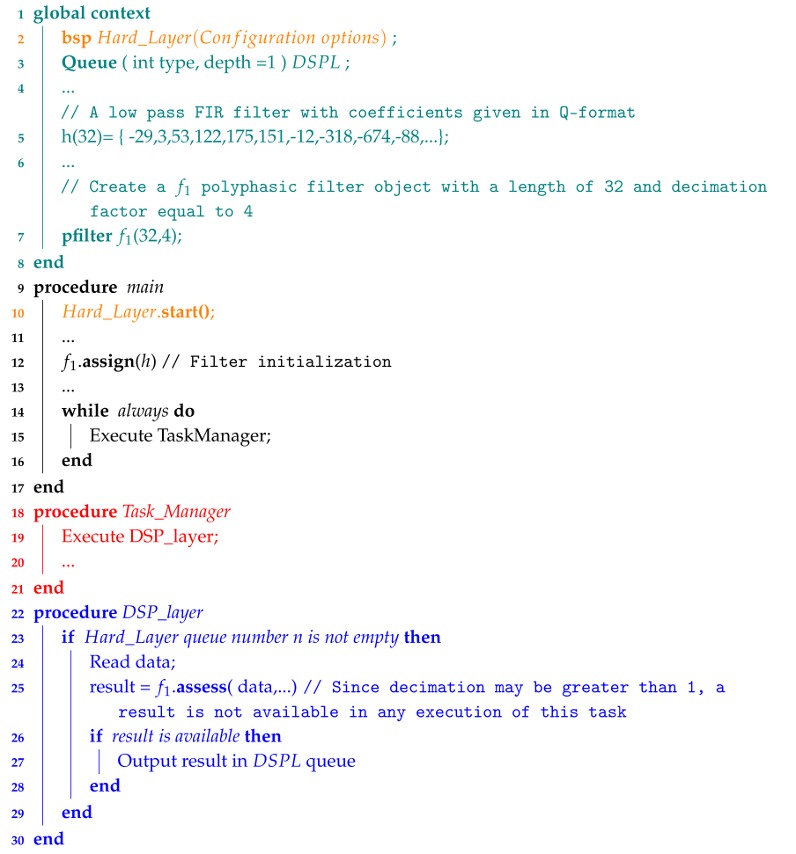


#### 3.2.3. Goertzel Class

Spectrum analysis is a very essential requirement in instrumentation and normally carried out by online or offline FFT processing. The Goertzel algorithm [48] is a digital signal processing (DSP) technique for identifying frequency components of a signal. While the general Fast Fourier transform (FFT) algorithm computes evenly across the bandwidth of the incoming signal, the Goertzel algorithm looks for a specific or predetermined frequency:

(3) $$s\left[n\right]=x\left[n\right]+2\cos\left(\omega_{0}\right)s\left[n-1\right]-s\left[n-2\right].$$

The first stage of the algorithm is based on the implementation the IIR filter given in Equation (3) where $\omega_{0}$ is the frequency to be analyzed. The output s[n] is iterated *N* times. In the last iteration, x[N] is set to 0. Then, power, *P*, is obtained by applying the Equation (4):

(4) $$P=s{\left[N-1\right]}^{2}+s{\left[N-2\right]}^{2}-2s\left[N-1\right]s\left[N-2\right]\cos\left(\omega_{0}\right).$$

Goertzel’s class has two methods: the *constructor*, which receives the $\cos(\omega_{0})$ and *N* as input arguments and sets the filter given by Equation (3) and *power* method, which receives a new data (x[n]), performs an iteration and computes Equation (4) in Q-format as the number of iterations equals *N*. Algorithm 5 shows a programming example illustrating the usage of the Goertzel class.

**Algorithm 5:** Programming example illustrating how to use the Goertzel class.
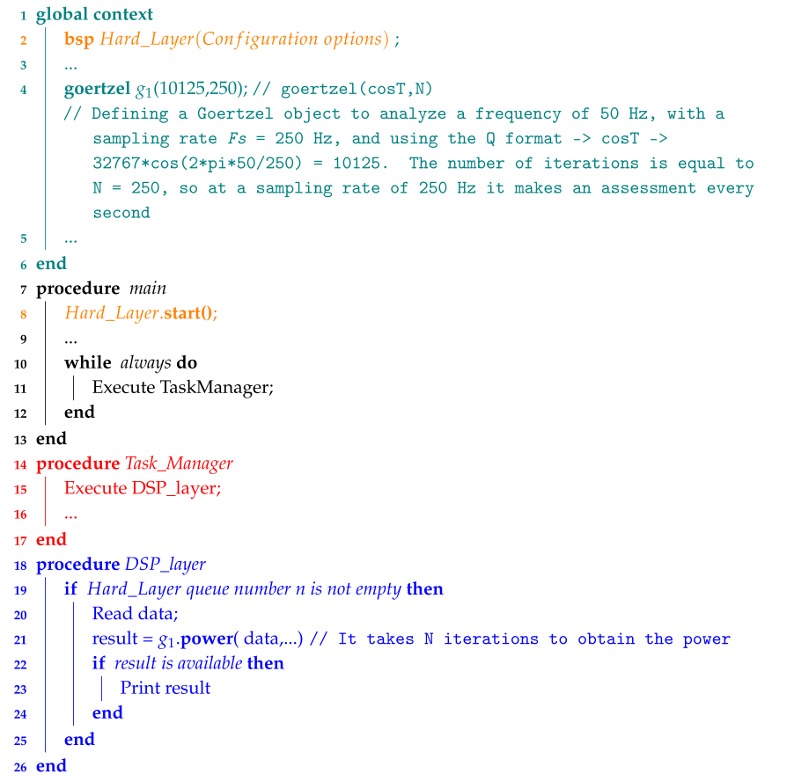


#### 3.2.4. Sliding Windows

In digital signal processing it is very common to collect a certain number of samples before applying a specific algorithm (FFT, auto-correlation, etc.). In real-time applications, samples are stored in a buffer and, when this is full, the algorithm to process it is called. This procedure is repeated continuously, collecting new blocks of data, which may, or may not, contain or share part of the samples of the previous blocks.

The technique of collecting blocks of data is known as *sliding windows*, where the two key parameters are: the number of data stored in a block, or length of the buffer (L), and the displacement or hop size (H), which represents the number of new samples that substitute the oldest ones. This technique reduces the time constraints for the DSP algorithm since it allows them to be executed in a time equal to $H/F_{s},$ which may be higher than the constrains for a digital filter ($1/F_{s}$).

Two classes have been developed to support the segmentation of incoming data in real time. The first one is the *segmentation* class, which has four main methods: *add*, which adds new data into the buffer, using the technique of circular buffering; *ready*, that returns 1 if the buffer is full or 0 otherwise; *access*, which enables access to an indexed element of the buffer; and *advance*, which makes it possible to empty the oldest H samples stored in the buffer.

The second class is *block*, which inherits from the segmentation class and is almost empty. Only a method to estimate the energy of a block of data has been provided. In this class, the programmer must write the algorithms that process the block of data.

To prevent data loss, the queue, which temporally stores samples from the hardware layer, must be correctly dimensioned. For example, if an algorithm took less than $H/F_{s}$ to execute, the displacement of the sliding windows and the size of the queue supplying data should be greater or equal to H samples. Algorithm 6 shows how to use the block class to obtain the energy of a block of 10 samples with a displacement of four samples.

**Algorithm 6:** Programming example illustrating how to use the block class.
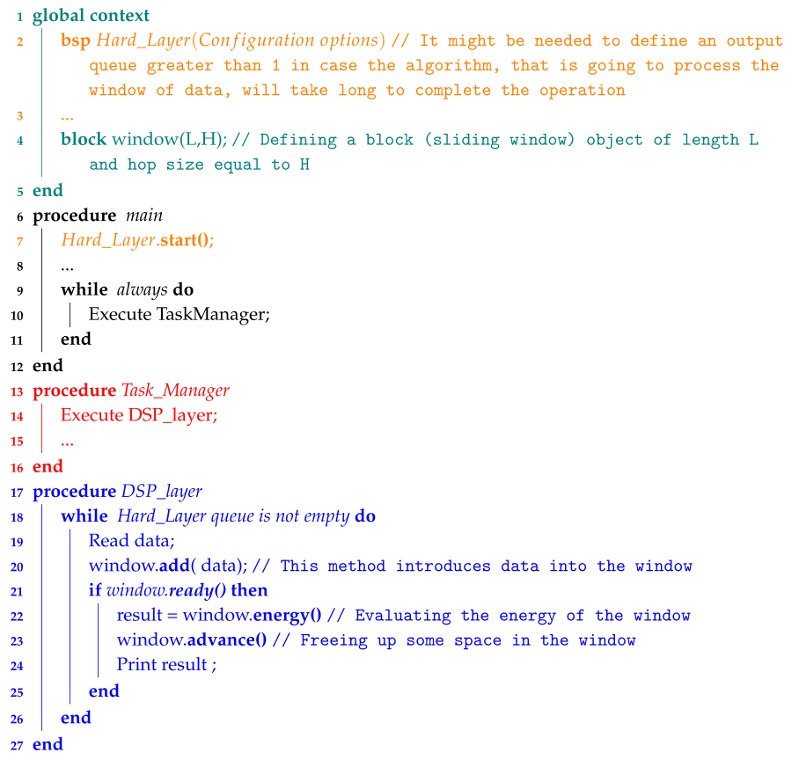


### 3.3. Application Layer

This layer hosts the set of functions that make the end-application. The programmer can split up this layer in others, if necessary, following the procedure proposed in this architecture. This is to include queues between adjacent layers and add the new functions in the Task_Manager. It is important that the execution does not stay in a single function for too long. For this reason, the use of long loops and delays must be carefully considered.

### 3.4. SWOT Analysis

To highlight the positive/negative aspects of the software architecture, a Strengths-Weaknesses-Oportunities-Threats (SWOT) analysis has been performed (Table 1). This kind of analysis allows for maximizing the strengths, taking advantage of their opportunities and overcoming their weaknesses [49]. The main weakness and threat of the software is the dependency it shows on the hardware platform until now. Nevertheless, having released the code under an open-source license and structuring it in several layers will allow programmers to develop new hardware layers for other platforms, besides increasing the functionality of the whole software.

## 4. Experimentation

**Figure 5:**
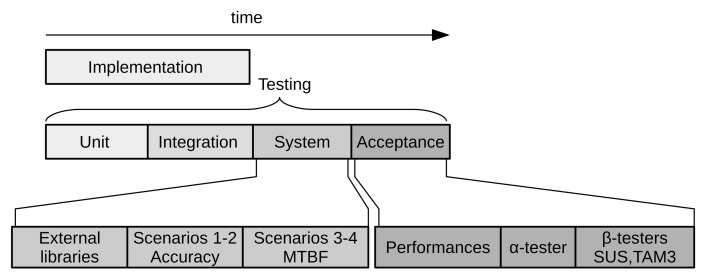
Methodology phases followed for software testing. During the implementation, the unit and integration phases were simultaneously accomplished. Then, in the system testing, the integration with external libraries and the evaluation of the library in different scenarios were carried out. Finally, the library performances were measured and sent to the α-tester who approved to proceed with the last part of the test in which some people (β-testers) performed two programming exercises and filled in several surveys (SUS, TAM3).

This section describes the methodology applied to implement, test and validate the proposed software. This methodology is depicted in Figure 5.

### 4.1. Implementation

The development and implementation of the software, together with the two first stages of testing, were simultaneously carried out. We chose the C++ programming language to implement the set of classes. This language generates efficient code, close to the assambler language, which is critical for digital signal algorithms. The Arduino IDE version 1.6.11 was used to integrate the classes with other high level libraries, and the Arduino/Genuino Uno board for testing. To generate the documentation, we used *Doxygen*, a free and Libre software under General Public License (GPL).

### 4.2. Unit and Integration Testing

During this first round of testing, the program is assessed to determine whether each component of the software (function, class, method) is fully functional, following the White-box Testing method [50].

Integration testing allows individuals the opportunity to combine all of the units within a program and test them as a group. This testing level is designed to find interface defects between the modules/functions.

### 4.3. System Testing

System testing is the first level in which the complete application is tested as a whole. The goal at this level is to evaluate whether the system has complied with all of the requirements.

We proposed testing the software in four different scenarios, named $SC_{1}$–$SC_{4}$. In the first two of them, we made an application that sends 5 min of raw data (input to DSP_layer) and processed data (DSP_layer output) through a serial port to a computer that stores it for further analysis. Then, we programmed the same DSP algorithm in Octave 3.0 (a GPL software compatible with Matlab) and applied it to the stored raw data. Both outputs, Octave and DSP_layer were compared by Pearson regression analysis.

- $SC_{1}$: **Blink detector**
  A system that allows people with disabilities to access a computer through the detection of a sequence of blinks/winks during a time interval. It consists of a glasses frame containing an infrared (IR) emitter/receiver placed close to the eyelid [45]. The IR receiver picks up lighting interference at 100 Hz when the eye is open. The frequency of this artifact is twice that of the power line frequency (100 Hz in our case), so that a double-zero low-pass filter, with coefficients b = [2048, 6628, 9458, 6628, 2048] and a = 26,810, was configured to remove it. The sampling frequency was set to 250 Hz.
- $SC_{2}$: **Estimating the power of a voice signal**
  In this second scenario, we want to obtain the power of a voice signal captured at 4 kHz. The sampled signals is underwent to a high-pass filter, which removes the bias component. The filter used was a Savitzky–Golay with coefficients [2731, −21,845, 0, 21,845, −2731] in Q15 format. Then, the signal was segmented using windows of 50-sample length, hop size of 25 and the power was obtained for each segment.

The last two scenarios tested the integration capabilities with other libraries, the use of more than one analog channel and the mean time between failures (MTBF). To do it, we programmed scenarios and let them run for eight hours. Data is sent to the computer, which counts the number of received samples and compares them with the correct number of samples according to the sampling frequency, number of channels, length of the data vector and decimation factor. The higher the MTBF, the higher the reliability of the system is.

- $SC_{3}$: **Frequency analysis with the FFT package**
  Here, we wanted to know the capability of this software when interacting with external packages like that which performs the FFT efficiently [43]. Namely, the FFT was applied to a one-channel stream of data sampled at 250 Hz and using 64 frequency bins. The block class was used to store segments of 64 samples and give more time to the FFT algorithm for completion. The hop size was equal to 64 samples.
- $SC_{4}$: **Smoothing/Decimation of the output of a two-axis accelerometer**
  This system needs to sample two channels simultaneously at a 250 Hz/channel and then apply a polyphasic 50-tap low-pass filter for smoothing and reducing the volume of information. The cutoff frequency is set at 5 Hz and the decimation factor is equal to 5. Here, we tested several channels and longer filter lengths.

### 4.4. Acceptance Testing

At the final level, acceptance testing is conducted to determine whether the system is ready to be released. Firstly, we analyzed the performances of each class. Then, the α-tester validated the work performed at that moment and allowed us to proceed with the final part of the experiment: the surveys.

#### 4.4.1. Performances

For each developed class, we tested the execution time, given in clock cycles and the memory usage, in bytes, both as functions that depend on several parameters such as filter length, decimation factor, etc. To achieve these results, we employed Atmel Studio 7.0 (Microchip Technology Inc., Chandler, AZ, USA), proprietary software that allows you to load, analyze and simulate assembler, C or C++ programs for the AVR microprocessor family.

#### 4.4.2. Usability

We wanted to find out the opinion of other professionals when using this software. Firstly, candidates had to have some basic knowledge of these research topics to be able to take part in the experiment. They were then asked to analyze the documentation and implement several applications involving the usage of digital signal algorithms. Finally, they were given a final test to rate the easiness/difficulty of different parts of the programming exercises and its usability in general.

##### Programming Exercises

Participants were given an Arduino Genuino with a shield containing an interface to a two-axis accelerometer and two LEDs, some programming examples illustrating most of the library methods and classes, general documentation and a list of the tasks grouped in two exercises described in Appendix A.

##### SUS and TAM3 Tests

In order to check the usability of the project, we made use of an already developed system denominated System Usability Scale (SUS) [51]. This is a system for the quick measurement of the usability of information technology (IT) systems that searches for errors so that they can be fixed. In recent years, many systems have been evaluated by SUS, proving its robustness, so the results obtained can be compared to previous studies.

As stated by the standard ISO 9241 [52], no specific property can be designated as usability, it therefore depends on the context. For this reason, the standard divides the measurement of usability in three parts:

- Effectiveness: depending on whether the users can achieve the objectives.
- Efficiency: the amount of resources required to achieve such objectives.
- Satisfaction: ease with which the objectives can be achieved.

Satisfaction is the easiest characteristic to compare between different systems or products as it is the least context dependent of the three. It is on this that SUS focuses [53], providing a measurement of subjective perceptions in an easy and quick way.

SUS consists of ten steps, each valued individually from one to five with alternating positive and negative states (Table A1 in Appendix B). The user needs to respond to the ten statements in a dynamic way. Let $x_{i}$ be the integer value ranging between 1 and 5 that a subject gave to statement $A_{i}$ in the SUS test. The global SUS score for such a subject is calculated by applying the Equation (5):

(5) $$Total\;raw\;score=2.5\times \left(\sum_{i\;evens}\left(5-x_{i}\right)+\sum_{i\;odds}\left(x_{i}-1\right)\right).$$

Our usability test included questions specifically chosen for our system (Table A2 in Appendix B) to assess how easy or difficult it is to implement a filter, a non-blocking delay timer, and configure the system to acquire a number of channels at a sampling frequency, etc. Additionally, the subjects answered some questions related to the time to complete each exercise or understand the documentation, suggestions for future improvements, etc. for a better assessment of their experience (Table A3 in Appendix B).

The technology acceptance model (TAM) was first developed to predict individual’s *behavioral intention* (BI) when using a new information technology (IT). TAM is determined by two beliefs: perceived usefulness and perceived ease of use. Both are influenced by external variables such as system characteristics, social influences, individual differences, etc. TAM2 provided more detailed explanations for the reasons that users found a given system usefulness, that is, subjective norm, image, job relevance, perceived ease of use, etc. and how experience and voluntariness can modulate them. TAM3 [54] extends the use of new factors to know the causes of the perceived ease of use. All of these models are useful tools to have a deep comprehension of users’ behaviors and know the reasons why they consider a specific IT useful or easy to use. Table 2 and Table 3 describe the set of determinants for *perceived usefulness* and *perceive ease of use*, respectively. Table A4 in Appendix C shows the statements associated with each determinant. In TAM3, the BI is also influenced by Voluntariness (VOL) and Experience. Results were analyzed by SPSS 24.

### 4.5. Participants (β-Testers)

We recruited 11 people from the Department of Electronic Technology; they included lecturers, PhD and undergraduate students who fulfilled the following prerequisites: basic C++ programming and knowledge of digital signal processing. Most participants were experienced with Arduino boards (81.8%) and graduate (63.6%). Table 4 summarizes the profiles of the subjects who took part in the experiment.

## 5. Results

This section shows the results obtained after applying the methodology described above. Section 5.1 contains the measurements of the failure and correctness/accuracy tests, while Section 5.2, Section 5.3 and Section 5.4 include the performances, usability and TAM3 tests, respectively.

### 5.1. System Test Results

**Figure 6:**
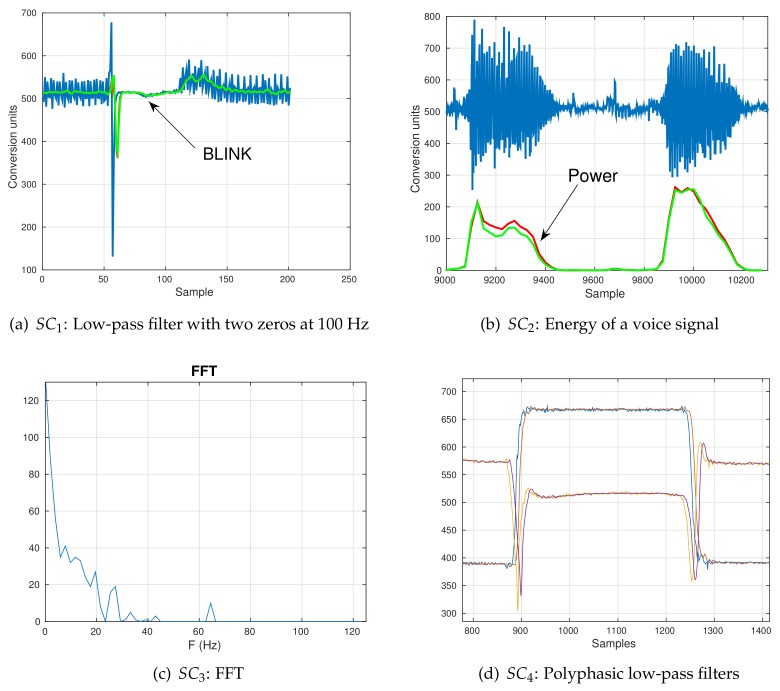
Output results after applying different signal processing techniques: (**a**) $SC_{1}$: a double-zero low pass filter to remove lighting interference at 100 Hz ($F_{s}$ = 250 Hz); (**b**) $SC_{2}$: an audio signal acquired at 4 kHz and its energy obtained by using a block class of length 50 and hop size equal to 25; (**c**) $SC_{3}$: the FFT applied to a one-channel stream of data at 250 Hz and 64 frequency bins; (**d**) $SC_{4}$: two 50-tap polyphasic low pass filters with a decimation factor of 4 applied to data sent by a two-axis accelerometer ($F_{s}$ = 250 Hz).

In this test, the correct operation of the whole software was checked. Figure 6 shows some output segments obtained in the proposed testing scenarios.

Figure 6a shows a noisy signal coming from an infrared (IR) LED mounted on glasses worn by subjects to detect blinks or winks ($SC_{1}$). The output of the filter running in the hardware is plotted in red and the one obtained in Octave in green. The Pearson correlation coefficient was equal to 0.982.

The output results and the voice signal from scenario $SC_{2}$ are shown in Figure 6b. The power signals were scaled by 50 in order to include the voice signal in the same plot. The Octave result is shown in green while the library output is shown in red. Both signals are almost identical, with a Pearson correlation coefficient of 0.971.

Figure 6c shows the FFT of a signal by including the FFT library configured to send 64 frequency bins of a signal sampled at 250 Hz (Scenario $SC_{3}$). There were no errors, so MTBF=∞.

In Figure 6d, two polyphasic 50-tap low-pass filters with a decimation factor of 4 were applied to smooth the data coming from a two-axis accelerometer. The delay can be seen between the smoothed filter outputs and the input raw signals due to the length of the digital filter. This experiment was run for 8 h and there were no errors, which means that the MTBF=∞.

All of these examples show that the software worked properly in different situations, sampling rates and when several channels were simultaneously being acquired.

### 5.2. Acceptance Test Results: Performances

In this section, we analyze the performances of the set of classes included in the software architecture using as metric the memory size in bytes and the number of clock cycles for their execution. Table 5 summarizes the results.

#### 5.2.1. Hardware Layer

The hardware layer contains a set of configuration functions (for timers 0,1 and ADC) and interrupt routines to support the non-blocking delays and the data acquisition. Configuration functions are only called once at the beginning, when the computer is turned on, so the time they take in executing is unimportant. However, Timer0 interrupt is called every 1 ms and Timer1 and ADC interrupts are called periodically depending on the established sampling rate. Therefore, it might be interesting, for the developing of real-time application, to find out how long those routines take to execute. This includes the number of cycles in executing the Timer0 interrupt when there are no active non-blocking delays, updating the Timer1 and ADC registers, reading the 2-byte data and pushing it into the specific channel queue (see Figure 3).

The number of cycles is roughly constant for the interrupt routines, although there is variable dependency on the depth of channel queue, $l_{q}$ (Equation (6)). When the pointers used to manage the data stored in such queues reach the ending address of the memory assigned to them, they need to be updated to the initial memory address. This operation spends some additional clock cycles. Therefore, the worst scenario would happen when the queue depth is equal to 1. Then, pointers must be updated every time new data is placed in the queue. As the queue depth increases, the number of clock cycles to execute this layer decreases on average according to the following relationship:

(6) $$\bar{C^{hl}}=\frac{10}{l_{q}}+87.$$

The maximum number of cycles, $C_{max}^{hl}$, is 97 for $l_{q}=1$.

The size of the program memory (PM) to allocate Hardware Layer routines is $PM_{size}^{hl}$ = 1934 B or, approximately, 6% of the whole PM. The size of data memory (DM) is variable and depends on how many channels, $N_{ch}$, have been configured for acquisition Equation (7). As each channel needs its own queue, and the queue class also needs some memory to allocate their pointers and internal variables, the total data memory size is given by the following relationship:

(7) $$DM_{size}^{hl}=4+\left(24+2l_{q}\right)\times N_{ch}.$$

In the equation above, the leftmost placed constant represents the size of the variables used in interrupt routines. The number 24 is associated with the internal variables in the queue class, and the term $2l_{q}$ the memory allocated to store the integer-size data in the queue.

#### 5.2.2. Filter Classes

In this section, we analyze the performances of three filter classes, which are implemented based on the first of them.

##### Filter Class

The two most important methods of filter class are those that allow programmers to assign coefficients (a[k],b[k] in Equation (1)) and assess filter output. The former is called once, during the booting-up, and it will not be considered in our analysis. The latter is called every time new data is available and knowing the execution time is important in real-time applications.

As seen in Section 3.2.1, the length of the filter, (p+q+2), depends on the number of coefficients it has in the causal part (p+1 coefficients) and anti-causal part (q+1) of Equation (1). We have analyzed the number of clock cycles used by the assess method of the filter class for different filter lengths (from 15 up to 254). We have not tested longer filter lengths because the class limits the size of causal/anti-causal part in order not to exceed 255 different coefficient (p,q<254) due to the reduced data memory size in Arduino Genuino boards. Up to 10 repetitions of each tested filter length were carried out. In each repetition, the coefficients were randomly generated to obtain the ${\bar{C}}^{fc}$ and $C_{max}^{fc}$ (Equation (8)), and the input sequence was also randomly generated. Our results show that a linear relationship exists between the number of cycles and the filter length ($R^{2}=0,99998$):

(8) $$C_{max}^{fc}\approx \bar{C^{fc}}=669+83.5\left(p+q\right).$$

There was a small difference between the $C_{max}$ and the average ${\bar{C}}^{fc}$ less than five clock cycles. The filter class requires 996 B of the PM and a space in DM given by the following relationship (Equation (9)):

(9) $$DM_{size}^{fc}=20+2\times \left(p+q+2\right).$$

The independent term is related to the internal variables used by the class. The remainder is employed to store the input/output terms (y[n−k], x[n−k]) that appears in Equation (1)).

Filter class also needs *p* + *q* + 2 coefficient as was said above, but the filter class does not store them internally. It has two pointers to a matrix containing such coefficients. Therefore, the memory used to store them is not part of the class, although it must be taken into account when computing the global resources of the whole software application.

##### Polyphasic Filter Class

The polyphasic filter class implements an FIR filter, based on building an array of D filter classes, where D is the decimation factor. This class also contains two main methods: assign and assess. Only the execution time of the assess method is taken into account in this study. When the assess method is called with new input data, the computation only takes part in one of the D filter classes. Every D input data, the filter output is returned. This means that the computation time of the polyphasic filter should be similar to the filter class but scaled by a factor of 1/D.

The length of the polyphasic filter is p+1, which corresponds to the causal part of Equation (1), and is bound to an upper limit of 255 terms. Experimentally, we have tested different filter lengths ranging from 16 up to 64, and a decimation factor ranging from 1 up to 32 at a power of 2. The coefficients and the inputs are randomly generated, following the same procedure as described above. The linear regression is then applied, which gave us the following relationship (Equation (10)):

(10) $$C_{max}^{pc}\approx \bar{C^{pc}}=772.5+83.6\frac{p}{D}.$$

As the decimation factor increases, the number of clock cycles decreases. The above equation is correct as the length of the filter is greater than the decimation factor (p+1>D); otherwise, the number of clock cycles would reach a constant value that is not affected by the decimation magnitude. This relationship is fairly similar to the one obtained in the analysis of the filter class.

The room in the PM to allocate the polyphasic filter code is of 626 B plus an additional 996 B in case the filter class has not been previously loaded in memory. The DM size depends on the filter length and decimation according to the Equation (11), where function ⌈.⌉ returns the upper integer of the input argument:

(11) $$DM_{size}^{pc}=10+D\times \left(20+4\times \lceil \frac{p+1}{D}\rceil \right).$$

##### Goertzel Class

The Goertzel algoritm uses the filter class to evaluate the output of an IIR filter given by Equation (3), which is tuned to the frequency to be detected. For *N* iterations, the Goertzel class only assesses the output of the IIR filter, and then the power is obtained by applying Equation (4). The *power* method performs these two operations (filter output assessment and energy calculation) spending a number of clock cycles given by Equation (12):

(12) $$\bar{C^{gc}}=965+\frac{821}{N+1}.$$

To obtain the equation above, we followed the same criteria as in previous experiments: the signal supplied was randomly generated and the experiment was repeated 10 times. The worst scenario happens when N=0; then, the number of cycles is $C_{max}^{gc}=1786$.

The Goertzel class only needs 55 bytes of PM (and an additional 996 bytes in case the filter class has not been previously loaded in the memory). The data memory size is constant here and equals 62 B.

#### 5.2.3. Block Class

The block class has two methods for adding data to a window and knowing if a window has been filled and is ready for being processed. Both methods take between 10 and 68 cycles to be executed. The method that consumes more clock cycles is the energy, which, in turn, depends on the length of the window, *L*, according to the Equation (13):

(13) $$C_{max}^{bc}=\bar{C^{bc}}=653+51L.$$

The PM size for this class is 390 B and DM size depends on the window length as well (Equation (14)):

(14) $$DM_{size}^{bc}=12+2L.$$

#### 5.2.4. Non-Blocking Delays

Non-blocking delays use the Timer0 interrupt routine, the timer_list and timer classes. The number of clock cycles depends on two factors: the TIMER0_RES, or $T_{0r}$ for short, which is set at 10 by default (that means 10 ms) and the number of timers, $N_{t}$. Every time the interrupt routine is called, an internal counter is compared to the preset resolution, $T_{0r}$. In case of equality, the timers in the list are updated. The number of cycles employed to perform such periodic operations is five for the interrupt routine plus $43N_{t}+37$ for to update the timers. As timers are only updated once in $T_{0r}$ times, the average number of cycles is given by Equation (15):

(15) $$\bar{C^{nd}}=5\left(1-\frac{1}{T_{0r}}\right)+\frac{1}{T_{0r}}\left(43N_{t}+37+5\right).$$

For example, at the default Timer0 resolution, $\bar{C^{nd}}=4.3N_{t}+8.7$. In the worst case, when the timer resolution is 1, the number of clock cycles is $C_{max}^{nd}=43N_{t}+42$. Additionally, using the results derived from the analysis of the number of clock cycles, we obtained the timers’ accuracy, which gave us a deviation value of less than 4 μs.

With respect to the memory resources, the non-blocking delays require 148 B of PM and $7+6N_{t}$ B of DM.

### 5.3. Acceptance Tests Results: SUS and General Assessment

**Figure 7:**
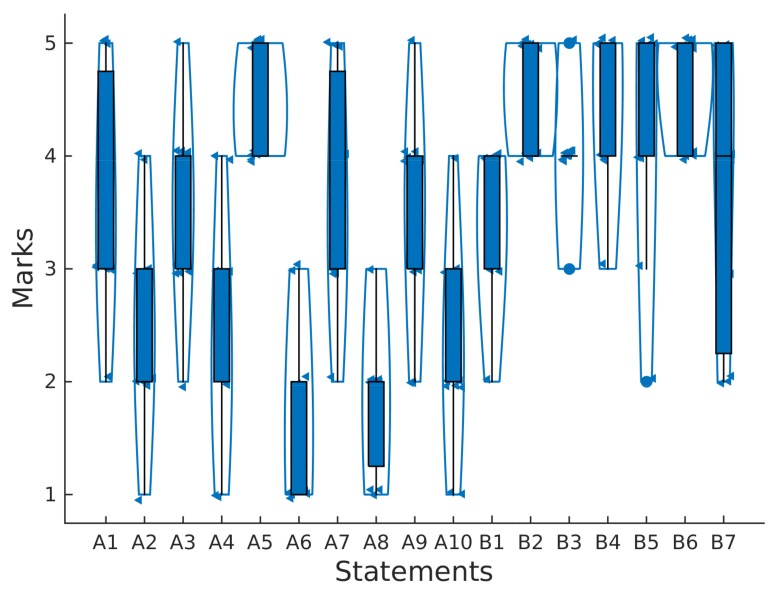
Box and violin plots containing the marks of SUS statements (A1, A2, ..., A10) and specific to the basic signal processing library (B1, B2, ..., B7).

Data collected in the usability test have been processed with Matlab as follows. Firstly, we have represented the descriptive statistics of the answers to questions shown in Table A1 and Table A2 for the SUS statements and for our specific library (Figure 7). In this figure, we can see violin diagrams containing all the data (small triangles), box plots and outliers (solid dots).

In general, even answers (A2, A4, ..., A10) have less marks than odd ones (A1, A2, ..., A9) as expected in good usability tests. Namely, the average SUS score for all participants was 70.2 out of 100, which means that the product is “almost good”. For participants S2, S3, the product was “excellent”, “good” for S4, S10, S11 and “ok” for the remainder.

The implementation of the proposed filters or the sliding window to obtain the energy was very easy for the participants, getting an average score of 4.6 4.4 and 4.6 for statements B4, B5 and B6, respectively. However, the implementation of non-blocking delays was more difficult, obtaining an average score of 3.7 out of 5 with higher variability among subjects, as can be seen in Figure 7.

**Figure 8:**
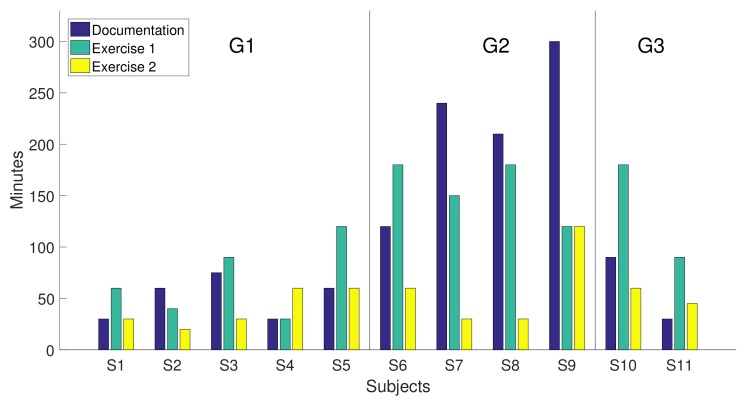
Time spent by participants to carry out the suggested activities.

Figure 8 shows the time spent by each of the subjects on the different parts of the experiment: the study of the documentation and the realization of the two programming exercises, each one represented with a bar. The order of the subjects on the *x*-axis is chosen according to groups shown in Table 4.

According to statements B1 and B3, the perception that the time spent to accomplish the exercises was ‘ok’ (4 on average) although it took longer to understand the documentation (3.4). In general, participants felt they could finish the programming exercise without external support (4.6).

To the question “*Write a general assessment of the software*”, the most common perception was that the software was easy and intuitive to use. About the documentation, they said it was well structured but that it could be improved by providing more examples.

For the last question “*Suggestions for future improvements*”, the subjects would like to have a more detailed explanation about the non-delay timers. Some of them proposed this software for other programming environments like Matlab or Octave as well as other platforms (like the ones based on ARM processors).

### 5.4. Acceptance Tests Results: TAM3

This analysis allows us to figure out the determinants that may, or not, influence the behavioral intention to adopt this new software. We excluded subjects belonging to group G2 from the analysis because: (a) they are undergraduate and not still integrated in a business or departmental environment; (b) the use/implantation of this software is being promoted in a university department, where it is more likely that people related to it can see the benefits or drawbacks of its use.

Subjects S5 and S11 did not fill in this survey, so the population under testing was reduced down to only five people, which limits the significance of the results. Table 6 shows the averages and standard deviations for all participants, and the Spearman coefficients between any two parameters, including its statistical significance.

As it can be seen, the *Behavioral Intention* (BI) obtained an average of 6.3 in a scale ranging from 1 to 7. It shows statistical dependency (ρ=0.918, *p* < 0.05) on *Perceived Usefulness* (PU) and *Perceived Ease of Use* (PEOU) (ρ=0.894, *p* < 0.05). This means that the intention to adopt this technology is mainly due to the fact that it is perceived as easy to use (PEOU = 5.55 in average) and useful (PU = 6.45) in general. Additionally, people perceived that the system is useful in part because the software is easy of use (ρ=0.975, *p* < 0.01).

## 6. Discussion

Filtering and frequency analysis are two important aspects in digital signal processing. The proposed software includes classes for both topics, allowing programmers to easily set up applications that might need to incorporate them.

### 6.1. Filtering

The $C_{max}$ and $DM_{size}$ parameters, explained in the previous section, provide valuable information that tells us to what kind of real-time applications the classes can be employed in when using an OSHW architecture like Arduino Genuino.

The filter class should be able to make a new output in less time than the period of time between samples. In other words, the number of cycles available, $C_{ava}$ for this algorithm to compute a new output must be equal to $F_{clk}/F_{s}$, where $F_{clk}$ is the clock frequency minus the number of cycles for the hardware class to acquire new data $C_{max}^{hc}$ (Equation (16)):

(16) $$C_{ava}=\frac{F_{clk}}{F_{s}}-C_{max}^{hc}.$$

**Figure 9:**
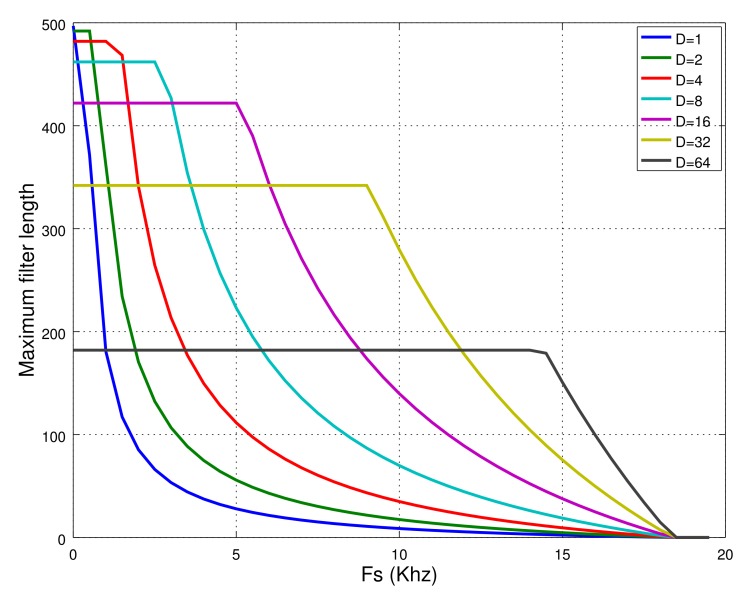
Theoretical maximum filter length as a function of the sampling rate ($F_{s}$) and decimation factor D. Each curve has been obtained by taking into account the room in the memory hosting the coefficients and the data, and the time available to compute the filter output as only one input channel is used.

Therefore, the $C_{max}^{fc}$ and $DM_{size}^{fc}$ parameters for the filter class must be less than the $C_{ava}$ and the whole size of the data memory. The maximum length of the filter must satisfy both of these conditions. Figure 9 shows the maximum length as a function of sampling rate and decimation factor when using only one analog channel.

The filter class limits the number of coefficients up to 256. This is because the coefficients and the data have been indexed by byte-size variables. By simply replacing them with integer-type variables, the length of the filter can be increased up to 64 k. In the discussion, we have preferred not to consider these library internal constraints and find out the computational limits of the class according to the memory usage and computation time. Therefor, such limits set a maximum filter length of 497 coefficients when using a sampling rate of 1 Hz, or 182 coefficients for a polyphasic filter with a decimation factor of 64 at a sampling rate of 14 kHz. As the decimation factor increases, so do the memory resources, which limits the length of the polyphasic filter as can be seen in Figure 9 for the lower sampling rate.

Figure 9 also shows that the maximum sampling rate for these classes when running in Arduino Genuino is theoretically set at 18.5 kHz. This rules out the possibility of using the filter class in Arduino for real-time audio applications. In fact, as explained in Section 3, we decided not to apply this software for audio applications in order to avoid reducing the resolution of the ADC. The Arduino Genuino platform limits the maximum sampling rate at 15 kHz for the maximum resolution of 10 bits. This fact forced us to use the integer size (16 bits) in the filter class, which is higher than the processor native data size. The filter class even uses long integer data for accumulating the sum of products given in Equation (1) and reducing overflow errors. All of these facts make the filter class take longer than if it had been built using the native 8-bit data size. In [42], the length of the convolution for a channel at a sampling rate of 31.25 kHz was analyzed. The results showed that it was only possible to implement a filter with a length of 1. However, if the integer division/multiplication could be replaced by shifting (in case of power of 2), the length of the filter could be increased up to 12–13. This could be a line to follow to improve the performance of the designed software. Nonetheless, the use of Arduino Genuino for real-time audio applications does not seem to be appropriate.

Other real-time applications need lower sampling rates than audio does. For example, authors in [55] show the minimal and optimal requirements for digital polysomnography, which includes a wide range of sources of measurement: electrocardiography (ECG), electroencephalography (EEG), body temperature, muscular activity (EMG), movement detection, oxygen saturation, breathing sounds, electro-oculography (EOG), etc. Most of them are under 250 Hz excluding the breathing sounds, whose optimal sampling rate is 5 kHz. Another example is shown in [56], which shows that the optimal sampling rate for human movement monitoring is 20 Hz.

For 250 Hz, the maximum filter length is roughly 500, which gives us a good idea of what kind of processing could be accomplished. In fact, one of the most common filtering applications in bioelectrical signals is a Notch filter, which just requires a small number of coefficients. A previous version of this software was used in the implementation of a notch filter with six coefficients to reduce the power line interference in the acquisition of an electrocardiogram signal [57]. With a similar filter length, this software has been used to implement a low pass filter with zeros at 100 Hz to guarantee the reduction of the lighting interference when using an infrared system to detect blinks [45]. The polyphasic filter class has also been used in [35] to apply a 64-tap low pass filter, with a decimation factor of 16, in order to smooth the three-axis data of an accelerometer in order to detect movements in children with cerebral palsy.

### 6.2. Frequency Analysis

There are two main aspects to be considered when performing frequency analysis. The first one is frequency resolution, ΔF, which depends on the length of the input data vector, *N*, and the sampling rate, $F_{s}$ according to the relationship: $\Delta F=F_{s}/N$. The second aspect is whether the algorithm used to compute the analysis will be able to perform the required operations in real-time. Both aspects are limited by both computational costs and data memory size.

**Figure 10:**
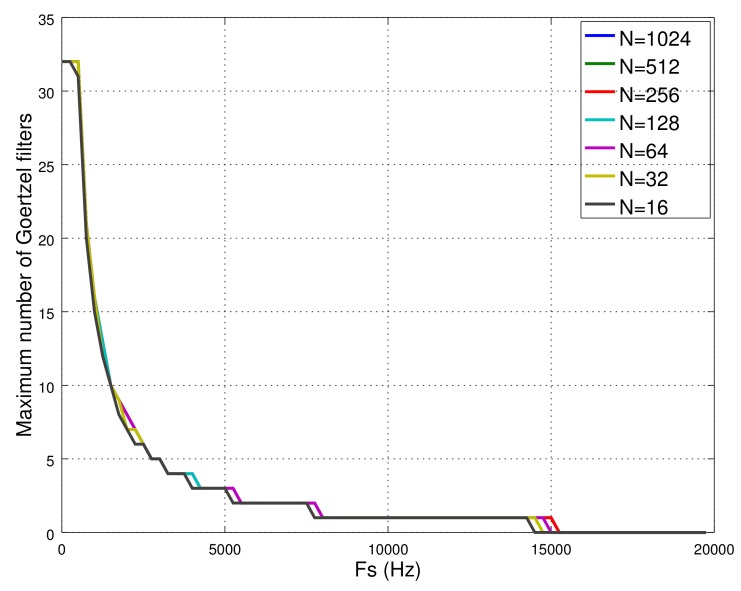
Theoretical maximum number of Goertzel filters as a function of the sampling rate ($F_{s}$) and number of iterations (N). Each curve has been obtained by taking into account the room in the memory hosting filter objects and the time available to compute them.

The developed software provides the Goertzel class, which makes it possible to measure the power of a specific frequency component of the input signal with a resolution given by the sampling rate and the number of iterations used in Equation (3). This algorithm does not need to store a vector of *N* input data. According to the results obtained in Section 5, this class uses 62 B of data memory, which limits the number of possible Goertzel filters to be operating simultaneously up to 32. Another factor limiting the number of Goertzel objects is the sampling rate, so that, as it increases, the time to compute the algorithm decreases (see Figure 10). Thus, with a sampling rate over 8 kHz, it is only possible to compute one Goertzel object, with none possible over 15 kHz. It is remarkable that the frequency resolution, in this algorithm, is given by the number of iterations. Therefore, it is possible to analyze, for example, the power of a frequency component of 1 Hz of a signal sampled at 10 kHz only by iterating the algorithm *N* = 10,000 times. Any frequency resolution is possible. Here, the cost is how long it takes the system to generate the output, which is the inverse of the frequency resolution.

There is an external library for computing the FFT of chunks of data (up to 256 bytes) for Arduino platforms [43]. We have analyzed the number of cycles and memory resources needed by this library. These results are summarized in Table 7.

The frequency resolution in the FFT depends on the length of the input vector, *N*. For example, at a sampling rate of 10 kHz and an input vector length of *N* = 256, the maximum frequency resolution would be ΔF≈39 Hz. During the computation of the FFT algorithm, it is necessary to store the following *N* input data. This can be done by the hardware layer class with a queue channel length of *N* elements. Obviously, this needs extra data memory room, which, in the worst scenario (*N* = 256), would be of 512 extra bytes. Table 7 shows that there is memory available to accomplish this. The number of clock cycles available to compute the FFT is given by Equation (17):

(17) $$C_{ava}=N\times \left(\frac{F_{clk}}{F_{s}}-C_{max}^{hc}\right).$$

This library has been optimized to compute the FFT algorithm very quickly. In fact, our results show that it is possible to assess an FFT with any *N* number of bins up to 10 kHz and with *N* = 16 bins up to 21 kHz. This means that such a library outperforms the Goertzel class in terms of the number of different frequencies that can be simultaneously analyzed and at higher sampling rates. This superiority is partly due to the fact that the library was implemented in assembler language, so its execution is faster, but with the certain drawbacks. These mean that: (1) its usage is strongly bound to the processor family; and (2) the number of the bins cannot be changed in execution time. Furthermore, the memory size per frequency is higher in Goertzel’s algorithm due to it needing to implement a filter.

The fact that the Goertzel class allows us to study certain frequency components with higher resolution frequency than the FFT library was what made us include this class in the software library.

### 6.3. Usability Test

The global assessment of the usability test gave an overall mark of “almost good” to the developed library after obtaining a score of 70.2 out of 100. Due to the average score obtained in the B1 statement (3.4), the fact that the time taken to study the documentation for G2 (around 200 min, which is much higher that the time spent by the other two groups around 50 min), and that more examples are required for future improvements, it seems that we need to revise the documentation and include more programing examples in the library. This might cut down the study time and speed up the implementation of applications.

**Figure 11:**
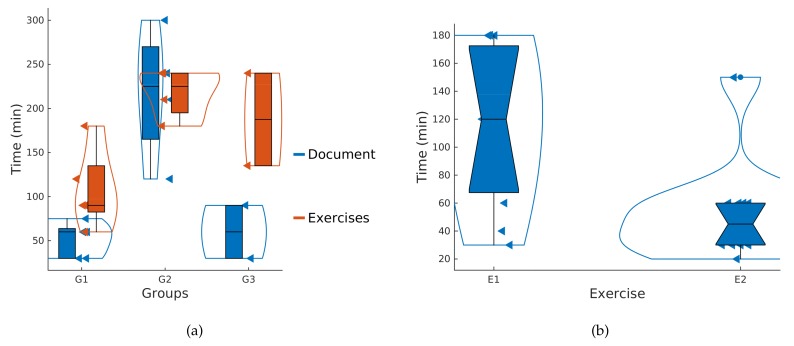
(**a**) completion time in reading the documentation and performing the exercises according to groups. (**b**) time to accomplish exercises 1 and 2 for all participants.

Figure 11a shows the boxplots containing the time spent studying the documentation and performing the two exercises for the three groups of participants. It is remarkable that G2 (undergraduate students) spent more time studying the documentation than the other two groups. This means that graduate students and lecturers caught up with the required knowledge very quickly (it was not necessary to perform any statistical test since the corresponding boxplots of G1 and G3 did not overlap with G2).

Another aspect is that lecturers were able to carry out the two exercises faster than the other participants (Wilcoxon rank sum test *p* = 0.013). Even though lecturers are familiar with Arduino IDE, this did not influence the time taken to perform the exercises. Groups G1 and G2 included people familiar with Arduino IDE, and the time spent by G2 was similar to the time spent by G3. In a formal comparison between experienced and non-experienced Arduino users, we did not find significant differences between the two groups (Wicoxon rank sum test *p* = 0.55).

Another aspect to highlight is the duration of the second exercise in comparison to the first one. Figure 11b shows the boxplots of the time spent by the eleven participants in both exercises. There is a statistically significant difference between the two groups (Kruskal–Wallis test *p* = 0.0085). This suggests that the learning and the usage of this library might be very fast. The time required to complete exercise 2 (40 min) was much less than that needed in exercise 1 (120 min).

Finally, to find out the specific reasons that make this technology suitable, we extended the Spearman correlation analysis among the items of the TAM3 survey. The *behavioral intention* statements show that people predict or will use/intend to use the system in the future (averages: $BI_{1}$ = 6, $BI_{2}$ = 6.4 and $BI_{3}$ = 6.6). There are relationships between $BI_{1}$ and $PEOU_{3}$ (ρ = 0.918, *p* < 0.05), $BI_{2}$ with $PEC_{2}$ (ρ = 0.889, *p* < 0.01) and $REL_{1}$ (ρ = −0.968, *p* < 0.01), and $BI_{3}$ with $PEC_{2}$ (ρ = −0.889, *p* < 0.01) and $REL_{1}$ (ρ = 0.968, *p* < 0.01). This means that people intend to use the software because they find it easy to use and predict/plan to use the software in the future because it is useful and there are enough resources to do it.

With respect to the *perceived usefulness*, we have found relationships between $PU_{3}$ (using the system enhances my effectiveness in my job, 6.4 in average) with $PEOU_{3}$ (ρ = 0.899, *p* < 0.05), $CSE_{1}$ (ρ=0.913, *p* < 0.05) and $PEC_{3}$ (ρ=0.913, *p* < 0.05), and $PU_{4}$ (the system is useful in my job, 6.4 in average) with $CSE_{4}$ (ρ=0.899,p<0.05), $CSE_{1}$ (ρ=0.889, *p* < 0.05) and $REL_{3}$ (ρ=0.913, *p* < 0.05). Putting all these determinants together, it can be concluded that the *perceived usefulness* comes from the fact that the system is easy to use and pertinent to the professional environment. Participants feel that given the necessary resources to use the software they will not need anyone around to help them complete the exercises.

Two statements in the *perceived ease of use* showed relationships with other determinants. Namely, $PEOU_{1}$ (the interaction with the system is clear and understandable, 5.4 in average) with $PEC_{1}$ (ρ=0.973, *p* < 0.01), $PEC_{2}$ (ρ=0.892, *p* < 0.05) and $SN_{2}$ (ρ=0.884, *p* < 0.01) while $PEOU_{4}$ (it is easy to get the system to do what the users want to do, 5.8 in average) with only $SN_{2}$ (ρ=0.884, *p* < 0.01). This means that the *perceived ease of use* mainly comes from the belief that users have control over using the system and the environment offers the material and human resources necessary to use the system.

## 7. Conclusions

This software allows programmers to implement basic signal processing applications easily in OSHW platforms like Arduino Genuino, one of the most popular, well-known, supported and inexpensive platforms.

The software includes a set of classes that implements filters, polyphasic filters, Goertzel’s algorithm, sliding windows, non-blocking delays and a hardware level class that guarantees that data are acquired at a fixed rate and delivered to a higher level of the software architecture through dedicated channel queues. Our results show that the library itself, together with a simple OSHW platform like Arduino Genuino, can be used for small biomedical real-time applications.

The product, which includes the library and documentation, obtained a score of 70.2 out of 100 in a standard SUS test. The implementation of typical signal processing elements was very easy and obtained a score over 4.4 on a scale with a maximum value of 5. Most participants perceived that the software was easy and intuitive to use, although it was also suggested that more examples should be included.

We will keep on improving the classes to make them more computationally efficient and exportable to other platforms such as those based on the ARM 32-bit processor, like Arduino Due, with higher data memory capacities, native data buses and clock frequencies than Arduino Genuino. This will widen the range of applications where the library can be used.

## Figures and Tables

**Table 1:** SWOT analysis applied to the software architecture

**Internal origin**	**Strengths**	**Weaknesses**
	• Open source • Documentation included • Multiple examples are available • Sampling frequency guaranteed by interruptions • Multiple analog sensors can be connected • Digital sensors can be sampled by timers • Cooperative multitasking • Layered architecture • Only one layer shows hardware dependency • Queue system to store and prevent data losing • Non-blocking delays to support loops and timing • Include most important topics in DSP algorithms	• The same $F_{s}$ (or submultiplies after software decimation) for all analog channels • The data length is limited up to 16 bits • The hardware layer depends on the platform • The number of filter coefficients in the casual and anti-causal parts is limited up to 255
**External origin**	**Opportunities**	**Threats**
	• Open source, which might rapidly increase the inclusion of new capabilities and ease its migration to other hardware platforms.	• The hardware layer depends on the platform

**Table 2:** Determinants for perceived usefulness.

Determinant		Definition
Perceived Ease of Use	PEOU	The degree to which a person believes that using an IT will be free of effort
Subjective Norm	SN	The degree to which an individual perceives that most people who are important to him think he should or should not use the system
Job Relevance	REL	The degree to which an individual believes that the target system is applicable to his or her job
Output Quality	OUT	The degree to which an individual believes that the system performs his or her job tasks well
Result Demonstrability	RES	The degree to which an individual believes that the results of using a system are tangible, observable, and communicable

**Table 3:** Determinants for perceived ease of use.

Determinant		Definition
Computer Self-Efficacy	CSE	The degree to which an individual believes that he or she has the ability to perform a specific task/job using the computer
Perception of External Control	PEC	The degree to which an individual believes that organizational and technical resources exist to support the use of the system

**Table 4:** Description of participant groups.

Group	Subjects	Experienced with Arduino	Level	Percentage (%)
G1	S1..S5	Yes	Graduate	45.4
G2	S6..S9	Yes	Undergraduate	36.4
G3	S10 and S11	No	Graduate	18.2

**Table 5:** Performances of the designed classes. Four parameters have been analyzed: program memory size, $PM_{size}$; data memory size, $DM_{size}$; number of clock cycles for some important class methods, on average, $\bar{C}$ or in the worst case $C_{max}$. (*) The $PM_{size}$ must be increased by 996 if the filter class has not been previously loaded in memory.

	Program Size	Data Size	Mean Execution Cycles	Max. Execution Cycles
Object	${\mathrm{PM}}_{\mathrm{size}}$ (B)/(%)	${\mathrm{DM}}_{\mathrm{size}}$(B)	$\bar{C}$	$C_{\max}$
Hardware Layer	1934/5.9	$4+\left(24+2l_{q}\right)N_{ch}$	$10l_{q}^{-1}+87$	97
Filter	996/3	20+2×(p+q+2)	669+83.5(p+q)	
Poly. Filter	626 */1.9	$10+D\left(20+4\lceil \frac{p+1}{D}\rceil \right)$	772.5+83.6p/D	
Goertzel	55 */0.2	62	$965+821{\left(N+1\right)}^{-1}$	1786
Block	390/1.2	12+2L	653+51L	
Non-Blocking Delays	148/0.5	$7+6N_{t}$	$5+37{T_{0r}}^{-1}+43N_{t}T_{0r}^{-1}$	$42+43N_{t}$

**Table 6:** TAM3 results. Columns M and SD show the average and standard deviation of all explored parameters, respectively. The Spearman correlation coefficient and its significance among the explored variables are also shown. (*) *p* < 0.05, (**) *p* < 0.01.

	M	SD	PU	PEOU	CSE	PEC	SN	VOL	REL	OUT	RES	BI
**PU**	6.4500	0.5104	1.000									
**PEOU**	5.5500	0.9987	0.975 **	1.000								
**CSE**	5.6000	1.3917	0.359	0.400	1.000							
**PEC**	4.8500	1.9808	0.718	0.800	0.500	1.000						
**SN**	6.3333	1.1127	0.379	0.369	0.105	0.738	1.000					
**VOL**	6.5333	1.0601	0.158	0.103	0.872	0.205	0.108	1.000				
**REL**	5.9333	0.8837	−0.296	−0.289	0.289	−0.577	−0.913 *	0.296	1.000			
**OUT**	6.4667	0.5164	−0.053	−0.051	0.872	0.205	0.108	0.947 *	0.296	1.000		
**RES**	5.0500	1.6694	0.918 *	0.894 *	0.112	0.783	0.648	−0.057	0.645	−0.229	1.000	
**BI**	6.3333	0.6172	0.918 *	0.894 *	0.224	0.447	0.000	0.000	0.000	−0.229	0.750	1.000

**Table 7:** Resources for computing the FFT algorithm given in [43]

N	Program Size	Data Size	Exec. Cycles
256	5004	1334	232554
128	4116	758	107338
64	3684	470	49560
32	3468	326	22860
16	3368	254	10793

## Appendix A. Programming Exercises

### Appendix A.1. Exercise 1

1. Set the sampling frequency to 250 Hz and configure the Arduino to acquire channels 0 and 1.
2. Program the DSP_layer function to send the incoming data through the serial port and visualize them using the Serial Port in Arduino IDE.
3. Apply a digital low-pass filter with a *Fc* = 25 Hz, length 25 to channel 0 of the accelerometer.
4. Apply a digital low-pass filter with a *Fc* = 10 Hz, length 50 to channel 1.
5. If the value of the filtered channel 0 is greater than 500, then the red led should be turned on and kept in this state 2 s after the level drops below 500.
6. If the value of the filtered channel 1 is greater than 500, then the green led should be turned on, otherwise turned off.

### Appendix A.2. Exercise 2

1. Set the sampling frequency to 1000 Hz and configure the Arduino to acquire only one channel.
2. Apply a Savitzky–Golay filter, which acts as a high-pass filter with coefficients h[n] = [2731, −21845, 0, 21845, −2731], to that channel.
3. Create a sliding window of length 30 and hop size equal to 5 to obtain the energy of the filtered signal.
4. If the energy is greater than 150, then the green led must be turned on for 5 s.

## Appendix B. SUS, Specific and Qualitative Questions in the First Survey

**Table A1:** SUS statements.

SUS Statements	Disagree - Agree				
**A1. I think that I would like to use this product frequently.**	1	2	3	4	5
**A2. I found the product unnecessarily complex.**	1	2	3	4	5
**A3. I thought the product was easy to use.**	1	2	3	4	5
**A4. I think that I would need the support of a technical person to be able to use this product.**	1	2	3	4	5
**A5. I found the various functions in the product were well integrated.**	1	2	3	4	5
**A6. I thought there was too much inconsistency in this product.**	1	2	3	4	5
**A7. I imagine that most people would learn to use this product very quickly.**	1	2	3	4	5
**A8. I found the product very awkward to use.**	1	2	3	4	5
**A9. I felt very confident using the product.**	1	2	3	4	5
**A10. I needed to learn a lot of things before I could get going with this product.**	1	2	3	4	5

**Table A2:** Specific statements.

Programing Exercise Statements	Disagree - Agree				
**B1. I did not require too much the to understand the documentation.**	1	2	3	4	5
**B2. I could finish the programming exercise without help.**	1	2	3	4	5
**B3. The time taken to make the application was OK.**	1	2	3	4	5
**B4. It was easy to implement a filter.**	1	2	3	4	5
**B5. It was easy to implement the Savitzky–Golay filter.**	1	2	3	4	5
**B6. It was easy to implement the sliding windows to obtain the energy.**	1	2	3	4	5
**B7. It was easy to implement the non-blocking delays to program the activation of the leds.**	1	2	3	4	5

**Table A3:** Qualitative questions.

Time taken to understand the documentation:
**Time taken to complete the exercises:**
**- Exercise 1:**
**- Exercise 2:**
**Write a general assessment of the software:**
**Suggestions for future improvements:**
**Date:**
**Signature:**

## Appendix C. TAM3 Statements in the Last Survey

**Table A4:** TAM3 statements.

	TAM3 Statements	Disagree - Agree						
**PU**	PU1 Using the system improves my performance in my job.	1	2	3	4	5	6	7
	PU2 Using the system in my job increases my productivity.	1	2	3	4	5	6	7
	PU3 Using the system enhances my effectiveness in my job.	1	2	3	4	5	6	7
	PU4 I find the system to be useful in my job.	1	2	3	4	5	6	7
**PEOU**	PEOU1 My interaction with the system is clear and understandable.	1	2	3	4	5	6	7
	PEOU2 Interacting with the system does not require a lot of my mental effort.	1	2	3	4	5	6	7
	PEOU3 I find the system to be easy to use.	1	2	3	4	5	6	7
	PEOU4 I find it easy to get the system to do what I want it to do.	1	2	3	4	5	6	7
**CSE**	I could complete the exercise using a library . . .							
	CSE1 . . . if there was no one around to tell me what to do as I go..	1	2	3	4	5	6	7
	CSE2 . . . if I had just the built-in help facility for in the documentation.	1	2	3	4	5	6	7
	CSE3 . . . if someone showed me how to do it first.	1	2	3	4	5	6	7
	CSE4 . . . if I had just the built-in help facility for in the examples.	1	2	3	4	5	6	7
**PEC**	PEC1 I have control over using the system.	1	2	3	4	5	6	7
	PEC2 I have the resources necessary to use the system.	1	2	3	4	5	6	7
	PEC3 Given the resources, opportunities and knowledge it takes to use the system, it would be easy for me to use the system.	1	2	3	4	5	6	7
	PEC4 The system is not compatible with other systems I use.	1	2	3	4	5	6	7
**SN**	SN1 People who are important in my professional environment think that I should use the system.	1	2	3	4	5	6	7
	SN2 In general, supervisors and workmates have been helpful in the use of the system.	1	2	3	4	5	6	7
	SN3 In general, the organization has supported the use of the system.	1	2	3	4	5	6	7
**VOL**	VOL1 My use of the system is voluntary.	1	2	3	4	5	6	7
	VOL2 My supervisor does not require me to use the system.	1	2	3	4	5	6	7
	VOL3 Although it might be helpful, using the system is certainly not compulsory in my job.	1	2	3	4	5	6	7
**REL**	REL1 In my job, usage of the system is useful.	1	2	3	4	5	6	7
	REL2 In my job, usage of the system is relevant.	1	2	3	4	5	6	7
	REL3 The use of the system is pertinent to my various job-related tasks.	1	2	3	4	5	6	7
**OUT**	OUT1 The quality of the output I get from the system is high.	1	2	3	4	5	6	7
	OUT2 I have no problem with the quality of the system’s output.	1	2	3	4	5	6	7
	OUT3 I rate the results from the system to be excellent.	1	2	3	4	5	6	7
**RES**	RES1 I have no difficulty telling others about the results of using the system.	1	2	3	4	5	6	7
	RES2 I believe I could communicate to others the consequences of using the system.	1	2	3	4	5	6	7
	RES3 The results of using the system are apparent to me.	1	2	3	4	5	6	7
	RES4 I would have difficulty explaining why using the system may or may not be beneficial.	1	2	3	4	5	6	7
**BI**	BI1 I intend to use it.	1	2	3	4	5	6	7
	BI2 I predict that I would use it.	1	2	3	4	5	6	7
	BI3 I plan to use the system in the future.	1	2	3	4	5	6	7
